# Effects of ischemic preconditioning on ischemia/reperfusion-induced arrhythmias by upregulatation of connexin 43 expression

**DOI:** 10.1186/1749-8090-6-80

**Published:** 2011-06-02

**Authors:** Zhenguang Chen, Honghe Luo, Mei Zhuang, Lie Cai, Chunhua Su, Yiyan Lei, Jianyong Zou

**Affiliations:** 1 Department of Thoracic Surgery, The First Affiliated Hospital, SUN YAT-SEN University, No. 58 Zhongshan Road 2, Guangzhou 510080, China; 2 Private Medical Center, The First Affiliated Hospital, SUN YAT-SEN University, No. 58 Zhongshan Road 2, Guangzhou 510080, China; 3 Department of Rehabilitation, The First Affiliated Hospital, SUN YAT-SEN University, No. 58 Zhongshan Road 2, Guangzhou 510080, China

**Keywords:** Cardioelectrial activity, Connexin43, Ischemic preconditioning, Myocardial hypertrophy

## Abstract

**Background:**

The susceptibility of hypertrophied myocardium to ischemia-reperfusion injury is associated with increased risk of postoperative arrhythmias. We investigate the effects of ischemic preconditioning (IP) on post-ischemic reperfusion arrhythmias in hypertrophic rabbit hearts.

**Methods:**

Thirty-three rabbit models of myocardial hypertrophy were randomly divided into three groups of 11 each: non-ischemia-reperfusion group (group A), ischemia-reperfusion group (group B), and ischemic preconditioning group (group C). Another ten healthy rabbits with normal myocardium served as the healthy control group. Rabbit models of myocardial hypertrophy were induced by abdominal aortic banding. Surface electrocardiogram (ECG) was recorded and Curtis-Ravingerova score was used for arrhythmia quantification. Connexin 43 (Cx43) expression was assessed by immunohistochemistry.

**Results:**

Ratios of heart weight to body weight and left ventricular weight to body weight increase significantly in the three groups compared with the healthy control group (p < 0.05). Arrhythmia incidence in group C is significantly lower than group B (p < 0.05). Curtis-Ravingerova score in group C is lower than group B (p < 0.05). Cx43 expression area in group A is smaller by comparison with the healthy control group (p < 0.05). Cx43 expression area and fluorescence intensity in group B are reduced by 60.9% and 23.9%, respectively, compared with group A (p < 0.05). In group C, Cx43 expression area increases by 32.5% compared with group B (p < 0.05), and decreases by 54.8% compared with group A (p < 0.05).

**Conclusions:**

The incidence of ischemia/reperfusion-induced arrhythmias in hypertrophic rabbit hearts decreases after IP, which plays an important protecting role on the electrophysiology of hypertrophied myocardium by up-regulating the expression of Cx43.

## Background

Various degrees of myocardial injury are present in hypertrophic hearts of patients undergoing open heart surgery. The hypertrophied myocardium differs from normal myocardium in myocyte architecture and myocardial blood supply. The decline of tolerance to ischemia-reperfusion injury of hypertrophied myocardium, due to pathological changes in cellular architecture and metabolism, is one of the causes of post-ischemic reperfusion arrhythmias. For the hypertrophied heart with a concomitant anomaly such as aortic valve stenosis, the vulnerability to arrhythmias increases with (1) a preoperative history of cardiac insufficiency and (2) insults of intraoperative hypothermic cardioplegia and ischemia/reperfusion.

The challenge in the management of postoperative arrhythmias lies in mastering the complexity of pathophysiology of arrhythmias. The derangement in the patterns of impulse conduction along the myocardium, especially in hypertrophied heart, is a factor contributing to the occurrence of postoperative arrhythmias. Peters *et al*. [1] showed that compared with normal adult human working ventricular myocardium, the surface area of gap junctions is reduced in ventricular myocardium from hearts subject to chronic hypertrophy and ischemia, which may induce abnormal impulse propagation in these hearts. Danik *et al*. [2] pointed out that Connexin 43 (Cx43), the predominant ventricular gap junction protein, is critical for maintaining normal cardiac electrical conduction, and its absence in the mouse heart results in sudden arrhythmic death. They insisted that the growing recognition that gap junction remodeling is a major contributor to the arrhythmogenic substrate in the diseased heart and suggested that uncoupling as a result of diminished Cx43 expression plays a mechanistic role in the formation of a highly arrhythmogenic substrate. Li *et al*. [3] found that decreasing gap junction plaque size was associated with increasing arrhythmogenecity in the absence of cardiomyopathy. And, N-cadherin function may be perturbed in diseased myocardium leading to altered gap junction organization thus generating an arrhythmogenic substrate.

In recent years, numerous studies related to myocardial protection have focused especially on ischemic preconditioning (IP), which is a phenomenon whereby brief periods of ischemia have been shown to protect the myocardium against a more sustained ischemic insult [4]. IP improves myocardial function and enhances myocardial tolerance to ischemia-reperfusion injury in part by triggering endogenous myocardial protection mechanisms including channels opening, attenuation of apoptosis, and proteins activation [5-8]. Myocardial protection against ischemia-reperfusion injury by IP has so far been well elucidated. It is imperative to dig deep into the protective role of IP on hypertrophied myocardium for which is more susceptible to ischemia-reperfusion injury than normal myocardium. A few studies have stated the beneficial effects of IP on hypertrophic hearts [9], however, the effectiveness and mechanisms have not yet been clearly demonstrated. Herein, we investigate the effects of IP on post-ischemic reperfusion arrhythmias in hypertrophic rabbit hearts.

## Methods

### Preparation of animal models of myocardial hypertrophy

Healthy New-Zealand rabbits weighing 2.3 ± 0.3 kg were provided by the Laboratory Animals Center of Sun Yat-Sen University with the approval of the local ethics committee. Rabbit models of myocardial hypertrophy were obtained according to the method by Gillis [10,11]. The rabbits were anesthetized with intravenous (marginal ear vein) injection of pentobarbital. Under sterile conditions, a median abdominal incision was made and the peritoneum was slit to expose the abdominal aorta. Finally the abdominal aorta was banded with the support of a hard catheter of 1.6 mm in diameter placed adjacent to it, to obtain a 40 to 60% stenosis. The rabbits were raised for six weeks until the day of sacrifice and then their hearts were extracted. Ratios of heart weight to body weight and left ventricular weight to body weight were calculated. The thickness of left ventricular free wall and interventricular septum was measured to assess the extent of myocardial hypertrophy. The heart extracted from healthy rabbit served as a control. The following criteria were necessary and sufficient for validating an animal model: (1) a 20% increase in myocardial weight and thickness, (2) Hematoxylin and Eosin (H&E) staining outlining hypertrophic myocyte morphology and alteration of intercalated disk structure (disruption and disorganization) [12].

### Experimental grouping

Thirty-three rabbits of myocardial hypertrophy were randomly divided into three groups of 11 each: non-ischemia-reperfusion group (group A), ischemia-reperfusion group (group B), and ischemic preconditioning group (group C). Another ten healthy rabbits with normal myocardium served as the healthy control group.

### Ischemia-reperfusion and ischemic preconditioning

During the process, heart rate and mean arterial pressures were recorded. Core body temperature was maintained at 37 °C with a thermo heating pad and monitored with the rectal thermometer. In group B, a 4-0 prolene suture with needle was passed through the myocardial surface below the left anterior descending coronary artery (LAD), and after the attainment of steady state heartbeat for 15 min, a 400 U/kg IV dose of heparin was administered and the LAD was ligated for 15 min to induce ischemia, with the support of a adjacent catheter. Thereafter, the myocardium was reperfused for 90 min. In group C, the LAD was ligated for three cycles of 5 min followed by 5-min reperfusion at first for IP, and the rest of the procedure was identical to that in group B.

### Measurement of electrophysiological parameters

After anesthesia, subcutaneous needle electrodes were inserted in all four limbs to record the surface ECG (lead II) using the BL-410 bio-functional experimental system. The ECG was recorded at 20-min intervals for 100 min and the mean value of ECG was used to determine the arrhythmia score in accordance with a modified Curtis-Ravingerova scoring system. In group A, parameters were measured right after the raising period. With PR or PQ segment as the isopotential line, an ST segment elevation or depression of at least 0.05 mV was considered as an abnormal ST-T segment change related to myocardial ischemia. A change of more than 20% in QRS duration was regarded as remarkable. The specific scoring was determined as follows: 1 point for ischemic ST-T segment changes, or supraventricular arrhythmia; 2 points for occasional ventricular extrasystole; 3 points for coupled ventricular extrasystoles, or ventricular extrasystoles in the form of bigeminal/trigeminal rhythm or more complex rhythm; 4 points for frequent ventricular extrasystoles (≥5 times/min); 5 points for ventricular tachycardia (VT) lasting less than 30 s; 6 points for VT lasting for at least 30 s; 7 points for VT with a period of several beats lasting more than 30 s; 8 points for ventricular fibrillation (VF) lasting less than 5 min; 9 points for VF with a period of several beats lasting less than 5 min or a VF lasting for at least 5 min; 10 points for VF with a period of several beats lasting more than 5 min [13].

### Determination and measurement of Cx43

Hearts of the rabbit were extracted and dried with a filter. Specimens of left ventricular myocardium were then collected in the region supplied by the LAD for HE staining and cytological examination. Cx43 was detected and measured using the immunohistofluorescence CY3 Kit (Boster Company). Specimens were frozen in liquid nitrogen and then fixed with acetone. After washing with PBS (phosphate-buffered saline), sheep serum was used to block non-specific antigens. Ten minutes later, drops of 1:100 dilution of polyclonal Cx43 antibody (Boshide Company) were added and the preparation was incubated overnight at 4 °C. After another wash with PBS, 1:60 dilution of biotin was added, followed by a second incubation at a stable temperature of 37 °C for 30 min. Drops of 1:120 dilution of fluorescein were then added. Finally, the preparation was mounted on a slide and preserved at 4 °C. Analysis and determination of the expression area and the fluorescence intensity of Cx43 was performed by confocal laser scanning microscopy. The analysis was completed by the assistance of the computer software. Acquired images were standardized by ignoring background pixels using the density slice manipulation. For semiquantitative analysis of Cx43 expression, the area and intensity of Cx43 immunopositive plaques were measured in a region (350 × 350 μm^2^), randomly selected from different areas.

### Statistical analysis

All statistical analyses were performed using SPSS (version 11.0). Data are presented as mean ± SD; we used t-test and one-way analysis of variance to assess differences between the above-mentioned groups. Statistical significance was set as 0.05.

## Results

### Establishment of animal model of myocardial hypertrophy

Ratios of heart weight to body weight and left ventricular weight to body weight, as well as left ventricular free wall thickness and interventricular septal thickness in group A significantly increase by 29.8%, 31.6%, 22.9% and 19.5%, respectively, in comparison with the healthy control group (P < 0.05) (Table 1). In addition, an obvious hypertrophic HE staining pattern in cardiomyocytes confirmed the successful establishment of animal model of myocardial hypertrophy.

**Table 1:** General data of healthy myocardium and hypertrophied myocardium

	Healthy control group	Hypertrophied myocardium
HW/BW	1.88 ± 0.16	2.40 ± 0.28**
LVW/BW	1.33 ± 0.12	1.75 ± 0.24*
Thickness of LVFW (mm)	4.20 ± 0.80	5.16 ± 0.75*
Thickness of IVS (mm)	3.90 ± 0.59	4.66 ± 0.66*

*P < 0.05, **P < 0.01, compared with the healthy control group (T test). HW: heart weight, BW: body weight, LVW: left ventricular weight, LVFW: left ventricular free wall, IVS: interventricular interventricular septum.

### Microstructure changes in myocardial cells

**Figure 1:**
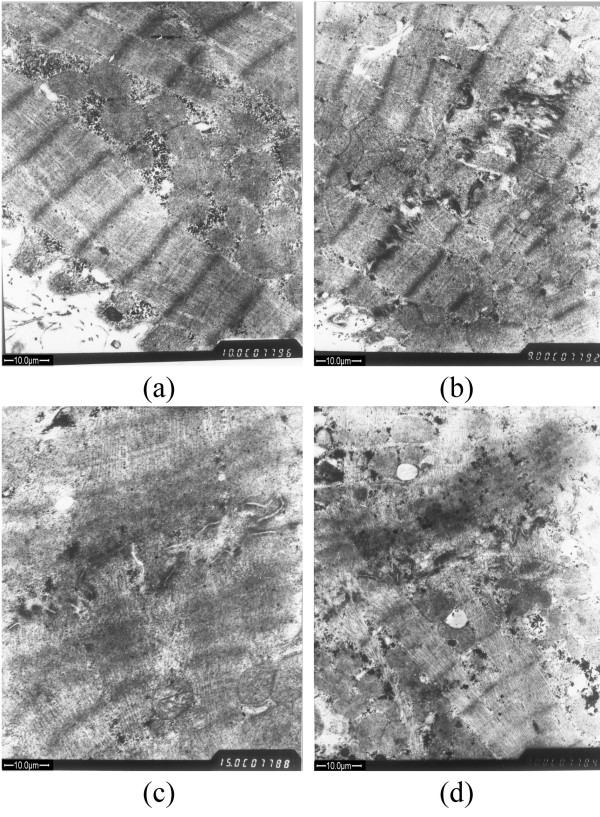
**TEM micrographs of myocardium**. (a) the healthy group; (b) group A; (c) group B; (d) group C.

Observation of healthy myocardium under electron microscope reveals normal continuous intercalated disks (Figure 1a). In group A, the discontinuity and disorganization of intercalated disks can be observed (Figure 1b). In group B, intercalated disks are disrupted in structure, and some are partially or even totally ruptured and disintegrated (Figure 1c). In group C where the myocardium sustains the same degree of ischemia-reperfusion injury after IP, structure of intercalated disks are significantly less distorted compared with that in group B (Figure 1d).

### Changes in cardioelectrophysiology in hypertrophied myocardium

In group B and C, ten minutes after LAD ligation, cyanosis is visible in the blood-supply region of the LAD. The ST segment becomes progressively elevated and stabilized afterwards. Cyanosis disappears and ST segment elevation resolves during myocardial reperfusion. In group B, the occurrence of ventricular arrhythmias is more serious than in group C. In group C, incidences of ventricular tachycardia, frequent ventricular extrasystoles and ST elevation are significantly lower than in group B (Table 2). The Curtis-Ravingerova score in group C (2.286 ± 1.380) is significantly lower than in group B (4.286 ± 1.976) (P < 0.05).

**Table 2:** Influence of ischemic preconditioning on arrhythmia incidence of hypertrophied myocardium

Incidence (%)	Ischemia-reperfusion group	Ischemic preconditioning group
Ventricular tachycardia	33.3	11.1*
Ventricular extrasystole	55.6	55.6
Frequent Ventricular extrasystole	22.2	11.1*
ST segment elevation > 0.05 mV	77.8	55.6*
QRS amplitude increase > 20%	55.6	66.7

*P < 0.05, compared with ischemia-reperfusion group (T test).

### Changes of Cx43 expression in hypertrophied myocardium

**Figure 2:**
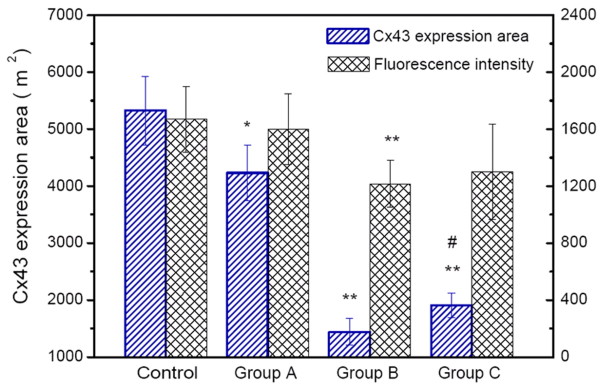
**Cx43 expression area and fluorescence intensity in rabbit myocardium measured by immunohistofluorescence**. *P < 0.05, compared with the control group; **P < 0.05, compared with group A; ^#^P < 0.05, compared with group B (one-way ANOVA). Control: healthy rabbit myocardium, Group A: non-ischemia-reperfusion group of hypertrophied myocardium, Group B: ischemia-reperfusion group of hypertrophied myocardium, Group C: ischemic preconditioning group of hypertrophied myocardium.

Cx43 expression area in healthy rabbit myocardium is 5325.62 ± 598.90 μm^2 ^and its fluorescence intensity is 1668.14 ± 231.16. Cx43 expression area in group A (4232.33 ± 484.43 μm^2^) is reduced by 20.5% compared with the healthy control group, but there is no significant difference in the fluorescence intensity (1599.43 ± 246.52). In group B, Cx43 expression area (1443.35 ± 231.46 μm^2^) decreases by 65.9% (P < 0.05) and the fluorescence intensity (1217.14 ± 162.44) is reduced by 23.9%, compared with group A (P < 0.05). In group C, Cx43 expression area (1911.72 ± 214.77 μm^2^) increases by 32.5% (P < 0.05) in comparison to that in group B, but is reduced by 54.8% (P < 0.05) when compared with group A. There is no significant difference in the fluorescence intensity between group B and group C (1301.00 ± 334.88). Curtis-Ravingerova score (Y) is significantly negatively correlated with Cx43 expression area (X) (r = -0.683, P < 0.05; regression equation Y = 10.137 - 4.08 × 10^-3^X, P < 0.05). Curtis-Ravingerova score and Cx43 fluorescence intensity are not correlated (Figure 2).

## Discussion

It is known that arrhythmia occurrence increases with the susceptibility of hypertrophied myocardium to ischemia-reperfusion injury during open heart surgeries [9,14,15]. Pathological changes in cell structure and metabolism of hypertrophied myocardium decrease its tolerance to ischemia-reperfusion injury. This happens especially in subjects with preoperative cardiac insufficiency, in which arrhythmias are more easily induced by stress and injury from intraoperative hypothermic cardioplegia and ischemia-reperfusion [16].

Various mechanisms of reperfusion arrhythmias in hypertrophied myocardium have been proposed [17,18]. In this study, intercalated disks of myocardial cells in hypertrophied rabbit myocardium appear disrupted and some partially or even totally ruptured and disintegrated after ischemia-reperfusion. Accordingly, ventricular arrhythmia occurrence increases with the degree of structural damage in the intercalated disks.

Applying IP to hypertrophied hearts is still a matter of controversy, as protective efficacy of IP has not yet been proved for hypertrophied myocardium. Some reports suggested that IP itself doesn't show direct antiarrhythmic effects, but delays or alleviates arrhythmias by reducing the necrotic area and delaying myocytes necrosis [19]. It has also been reported that the role of IP in the prevention of arrhythmias is related to its ability to change electrophysiological properties of myocardial tissues [13,20]. In this study, lower incidences of ventricular tachycardia and frequent ventricular extrasystoles are observed in hypertrophic rabbit hearts with IP before ischemia-reperfusion injury. Moreover, Curtis-Ravingerova score is reduced by approximately 50% and Cx43 expression area increases by over 30%. Curtis-Ravingerova arrhythmia score is negatively correlated with Cx43 expression area. There is no significant difference in Cx43 fluorescence intensity. Our results suggest that IP may reduce arrhythmia occurrence after ischemia-reperfusion by maintaining the spatial distribution of Cx43-based gap junction channels, and hence possibly protecting electrophysiological properties of myocardial tissues.

In addition, Cx43 expression area, an important architectural factor related to post-ischemic reperfusion arrhythmias in hypertrophied myocardium, is reduced by 20.5% compared with normal myocardium. However, there is no significant difference in fluorescence intensity. Cx43 expression area and fluorescence intensity in hypertrophied myocardium after ischemia-reperfusion are reduced by 65.9% and 23.9%, respectively, compared with non-ischemia-reperfusion hypertrophied myocardium. Similar results have been reported by other studies [21], suggesting the existence of an electrophysiopathological basis of arrhythmias after ischemia-reperfusion in hypertrophied hearts, which may be related to the fact that chronic myocardial hypertrophy leads to electrophysiological-related microstructural changes. These microstructural changes may be associated with the decline in Cx43 expression area, as well as the diminution in the number of low-resistance channels mainly composed of Cx43. Therefore, the slow myocardial electrical conduction and the prolonged cardiac repolarization make hypertrophied myocardium more vulnerable [22]. This is one of the risk factors for post-ischemic arrhythmias in hypertrophic hearts. The down-regulation of the fluorescence intensity of Cx43 after ischemia-reperfusion in hypertrophied myocardium suggests an alteration of the permeability of gap junction channels, which initiate action potential and possible after-depolarization activity and thus constitute another pathway leading to post-ischemic arrhythmias.

Results from Cx43 fluorescence intensity measurements suggest that the permeability of Cx43-formed gap junction channel is less affected by IP. In recent years, the activation of protein kinase (PKC) has been reported as an important element in myocardial protection with IP. It functions by promoting phosphorylation of a number of effective myocardial proteins, including connexin molecules, through signal transduction system [23-27,29]. Effective distribution of Cx43 molecules and the status of Cx43 phosphorylation are determinant factors of conductance and permeability of gap junction channels [30].

## Conclusions

Our study suggests that the incidence of ischemia/reperfusion-induced arrhythmias in hypertrophic rabbit hearts decreases after IP, which plays an important antiarrhythmic role in hypertrophied myocardium during ischemia-reperfusion by maintaining the integrity of its electrophysiological features such as the up-regulation in Cx43 expression area. As it is known that Cx43 can quickly translocate between several organelles under pathologic conditions such as ischemia, the changes in membrane connexin or gap junction plaque density should be further elucidated by other techniques such as RT-PCR, which will be reported in future work.

## Competing interests

The authors declare that they have no competing interests.

## Authors' contributions

All authors have read and approved the final manuscript. ZGC and HHL contributed equally to this work, both of them designed study, collected data, analyzed data, and wrote manuscript. MZ, LC, CHS, YYL, and JYZ analyzed data, and wrote manuscript.
